# Post-Traumatic Stress Disorder Following a Suicide Attempt

**DOI:** 10.1192/j.eurpsy.2025.1944

**Published:** 2025-08-26

**Authors:** S. Ben Aissa, R. Khouloud, N. Chaima, L. Amine, M. Wahid

**Affiliations:** 1 Psychiatry D, Razi Hospital, Mannouba, Tunisia

## Abstract

**Introduction:**

Post-traumatic stress disorder (PTSD) is an anxiety disorder that develops when a person is exposed to death, perceives a threat to their safety, or witnesses a traumatic event, either personally or vicariously. It is a condition that affects over 70% of adults who have experienced trauma at least once in their lives. What happens in the case of individuals who have attempted suicide? Do they also frequently develop PTSD?

**Objectives:**

To determine if individuals who have attempted suicide are at risk of developing PTSD as a result of their suicide attempt. If such a connection is proven, what therapeutic measures could be proposed to prevent the onset of this disorder?

**Methods:**

This is a descriptive study using a survey of patients at the Department of Psychiatry D at RAZI Hospital, who have made one or more suicide attempts during the year 2023.

**Results:**

20 patients (80%) exhibited a moderate to severe depressive episode at the time of their suicide attempt. The average number of suicide attempts was 1.53. The methods used for the suicide attempts included medication ingestion, observed in 15 patients (60%), phlebotomy in 4 (16%), jumping from a height in 2 (8%), hanging in 2 (8%), and ingestion of toxins in 2 (8%). 14 patients (56%) required hospitalization in a medical unit following the suicide attempt. 9 out of 25 patients (36%) developed PTSD according to DSM-5 criteria.

**Conclusions:**

A significant proportion of suicide attempt survivors may develop PTSD related to the suicide attempt. PTSD related to a suicide attempt could serve as a viable target for assessment and intervention to improve quality of life and reduce the risk of future suicide among individuals who have attempted suicide. However, more studies are needed to evaluate the risk of PTSD in this population.

**Disclosure of Interest:**

None Declared

